# Mechanisms of Hypertension in Women: Interactions Between Vascular Ageing, Metabolic Dysfunction, and Hormonal Regulation

**DOI:** 10.3390/biomedicines14081667

**Published:** 2026-07-24

**Authors:** Shiva Hooshmandi, Nicholas S. Freestone, Francesca I. F. Arrigoni

**Affiliations:** School of Life Sciences, Pharmacy and Chemistry, Kingston University London, Kingston upon Thames KT1 2EE, UK; n.freestone@kingston.ac.uk (N.S.F.);

**Keywords:** hypertension, sex differences, menopause, vascular ageing, endothelial dysfunction, renin–angiotensin–aldosterone system (RAAS), insulin resistance, nitric oxide, cardiovascular risk, hypertensive disorders of pregnancy

## Abstract

**Purpose:** Hypertension in women is a dynamic, hormone sensitive condition shaped by cumulative physiological changes across the life course. This review summarises current evidence relating vascular ageing, hormonal regulation, metabolic dysfunction, and reproductive history to blood pressure regulation in women. **Materials and Methods:** A narrative review of the literature was conducted using PubMed, Scopus and Google Scholar. Clinical, epidemiological, and mechanistic studies were synthesised to evaluate factors influencing hypertension in women. Reports in which menopausal status was not defined, or previous reproductive milestones were not documented, were excluded or interpreted with caution. **Results:** Evidence suggests that menopause, vascular ageing, metabolic dysfunction, androgen to oestrogen balance, and reproductive history interact to influence endothelial function, neurohormonal regulation, renal sodium handling, and vascular resistance. Ageing-related mechanisms, including cellular senescence, chronic low-grade inflammation, and genetic susceptibility, may contribute to increased cardiovascular risk. Hypertensive disorders of pregnancy identify women who are at higher risk of developing cardiovascular disease later in life and provide an opportunity for earlier risk assessment and prevention. Emerging therapies, including GLP-1 receptor agonists and SGLT2 inhibitors, may offer additional options for improving cardiovascular risk management, although their role in sex-specific prevention remains an evolving area of research. **Conclusions:** Hypertension in women is best understood within a life-course framework. Incorporating reproductive history, menopausal status, metabolic health, and emerging risk markers may improve cardiovascular risk assessment and support earlier intervention.

## 1. Introduction

Hypertension is a major component of the metabolic syndrome and one of the leading modifiable risk factors for cardiovascular disease and stroke worldwide [1]. Epidemiologically, men exhibit a higher prevalence of hypertension in early- and mid-adulthood. But women tend to maintain lower blood pressure until midlife, followed by a marked increase after the fifth decade that ultimately surpasses that of men in older age [2,3]. Systolic blood pressure increases across the menopausal transition. Interestingly, the accelerated rise in some women is not fully explained by chronological ageing alone [4,5].

This shift happens with changes in arterial stiffness, endothelial function, metabolic health, and hormonal status, suggesting that the transition from premenopausal to postmenopausal physiology is accompanied by dynamic changes in the blood pressure regulatory system [6,7].

Midlife systolic surge is linked to endothelial dysfunction and vascular ageing processes associated with oxidative stress and reduced nitric oxide bioavailability [6,7].

These changes do not occur in isolation. Declining oestrogen levels and age-related increases in oxidative stress contribute to the deterioration of endothelial signalling, promoting a shift toward a more oxidative vascular environment [8,9]. As reactive oxygen species accumulate, endothelial nitric oxide synthase becomes uncoupled, such that it generates superoxide rather than nitric oxide. This further disrupts vasodilatory capacity and reinforces the increase in vascular resistance that characterises midlife hypertension [6,10].

Persistent midlife hypertension is associated with cerebrovascular dysfunction, including impaired autoregulation, microvascular rarefaction, and potential alterations in blood–brain barrier integrity, which may contribute to increased risk of vascular cognitive impairment [11,12]. As systemic vascular changes influence cerebral autoregulation, the postmenopausal brain may be increasingly susceptible to microvascular injury and cognitive decline.

However, menopause should not be viewed merely as an isolated causal driver of late onset disease. Instead, this review defines menopause as a systems-level biological inflexion point that unmasks pre-existing vascular vulnerabilities, including subclinical microvascular damage and neurovascular dysregulation earlier in life [13,14]. In this review, vascular reserve refers to the capacity of the vasculature to adapt to physiological and pathological stressors while maintaining vascular function.

**Figure 1:**
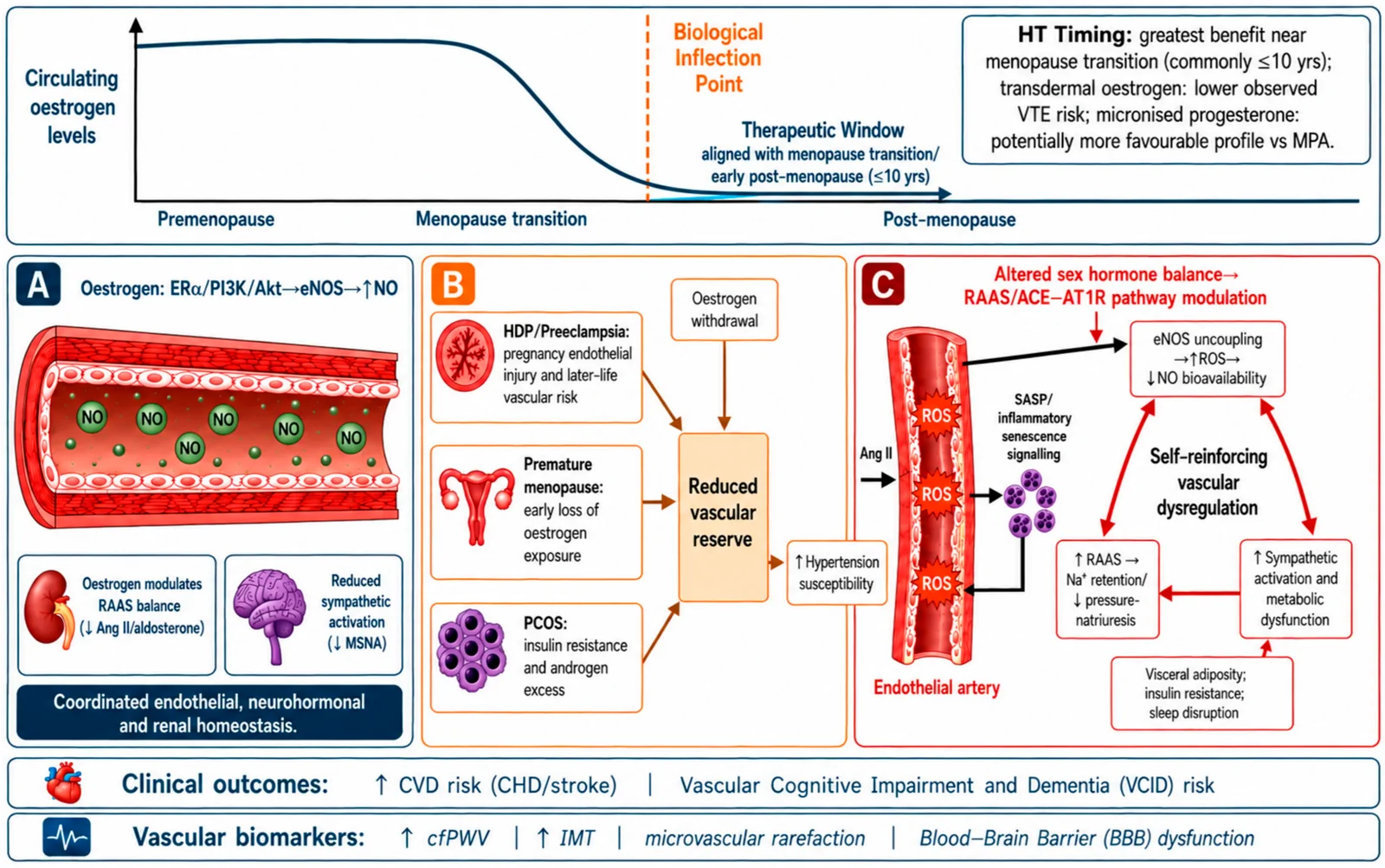
Integrated vascular mechanisms contributing to hypertension in women across the menopausal transition. (**A**) Before menopause, oestrogen supports vascular homeostasis through coordinated effects on endothelial nitric oxide signalling, neurohormonal regulation, and renal sodium handling. (**B**) Female-specific cardiovascular factors, including hypertensive disorders of pregnancy, premature menopause, and polycystic ovary syndrome, may reduce vascular reserve and increase susceptibility to hypertension. (**C**) Oestrogen withdrawal, altered sex hormone balance, inflammatory senescence signalling, metabolic dysfunction, and neurohormonal activation contribute to vascular dysregulation characterised by impaired nitric oxide bioavailability, oxidative stress, and RAAS activation, ultimately increasing hypertension risk and adverse cardiovascular and cerebrovascular outcomes. Created in BioRender. Hooshmandi, S. (2026) https://BioRender.com/na3g702, accessed on 19 July 2026.

We propose an integrated multi-level framework instead of traditional compartmentalised models. In this framework, the menopausal transition interacts with endothelial dysfunction, neurohormonal regulation, metabolic dysregulation, vascular ageing, and female-specific cardiovascular modifiers to influence blood pressure regulation [13,15]. Female-specific cardiovascular modifiers, including hypertensive disorders of pregnancy, premature menopause, and polycystic ovary syndrome, may further reduce vascular reserve and increase susceptibility to vascular dysregulation, consistent with evidence linking these conditions to adverse vascular and cardiovascular outcomes [16,17,18,19]. These interacting mechanisms contribute to increased vascular resistance and hypertension risk across midlife and later life [20,21] (Figure 1). This approach is also helpful for resolving the clinical controversies surrounding hormone therapy (HT). The cardiovascular benefits of HT are highly dependent on whether treatment is initiated within a specific window after menopause while the vascular lining remains healthy [22,23]. Modern delivery methods, such as transdermal oestradiol, have also been identified to offer a safer metabolic and blood pressure profile than traditional oral formulations [22,24].

Hypertensive disorders of pregnancy, including gestational hypertension and preeclampsia, are associated with persistent endothelial dysfunction, microvascular injury, and adverse vascular remodelling that may extend well beyond the peripartum period and are now recognised as early indicators of increased long-term cardiovascular risk [16,23].

A history of preeclampsia is associated with a specific long-term pattern where metabolic and hormonal changes during menopause combine with an already reduced vascular reserve [16].

Underlying endothelial and vascular abnormalities associated with hypertensive disorders of pregnancy (HDP) may remain clinically silent during early adulthood but have been linked to persistent microvascular injury and adverse vascular remodelling following pregnancy [16,25,26]. This can influence the rapid acceleration of systemic resistance and clinical hypertension. Clinical evidence also identifies premature menopause (age < 40), and chronic infertility conditions, as female-specific factors associated with increased cardiovascular risk [3,17]. These factors lower the threshold for vascular stiffening and cellular senescence through chronic, sterile inflammation [27,28]. 

Women with polycystic ovary syndrome (PCOS) have an increased risk of adverse cardiometabolic outcomes and hypertension. This association has been linked to insulin resistance, obesity, dyslipidaemia, hyperandrogenism, and endothelial dysfunction, which may contribute to increased cardiovascular risk later in life [18,19]. Within the framework proposed in Figure 1, PCOS may represent an early life metabolic and hormonal exposure that contributes to reduced vascular reserve and increased susceptibility to later cardiovascular dysfunction [18,19].

Although many pairwise interactions among endothelial, neurohormonal, autonomic, and metabolic pathways are supported by human and experimental studies [15,29,30], the framework proposed here should be interpreted as a conceptual synthesis of existing evidence, rather than a fully developed or experimentally validated systems-level model. The following sections examine these regulatory systems individually before integrating them into interacting pathways that reflect physiological regulation in midlife women.

## 2. Materials and Methods

This synthesis was developed from a structured survey of peer-reviewed research published between January 2000 and early 2026. PubMed and Scopus were used as databases. The primary aim was to integrate established clinical evidence with emerging concepts from vascular geroscience. To this end, we used search strings crossing “hypertension in women” with specific regulatory nodes. We incorporated descriptors of oxidative stress eNOS uncoupling, senescence-associated secretory phenotype (SASP), hepatic first pass metabolic effects, and sex-specific polygenic risk architecture. To satisfy the technical requirements for an integrated systems model, we established a strict hierarchy of evidence. We gave preference to robust human data from long-term cohort studies, such as SWAN and MESA [4,31]. A specific priority of this review was the characterisation of clinical risk factors associated with hypertension. Therefore, our search parameters were expanded to include a wide range of biological and environmental modifiers of both preeclampsia and chronic hypertension. Evidence regarding maternal age, adiposity, vascular ageing, and the influence of pre-existing comorbidities such as chronic kidney disease were specifically targeted, and studies in which menopausal status was not defined, or reproductive history was not reported, were given lower priority [31].

We applied specific physiologic filters to account for the discrepancies in the clinical literature, especially around hormonal interventions. We harmonised inconsistent results by sorting data by delivery route (transdermal vs. oral) and time of commencing treatment (timing hypothesis) [22]. For key risk nodes, we used meta-analyses of large-scale GWAS and polygenic risk architecture [32]. Animal and in vitro studies were used as secondary evidence to support mechanistic interpretation and complement findings from human studies, particularly for intracellular pathways involving oxidative stress and nitric oxide signalling. We limited these to the explanation of intracellular pathways, such as oxidative stress and nitric oxide signalling pathways [6]. The objective of bringing these diverse data sets together is to move the literature from a sequential list of risk factors to an integrated systems-level model.

## 3. Vascular Tone

Arterial blood pressure is determined by vascular tone, which reflects the combined influence of endothelial signalling, neurohormonal control, renal sodium handling, and hemodynamic forces acting on vascular smooth muscle [33,34].

### 3.1. Endothelial Regulation and Nitric Oxide Signalling 

Nitric oxide (NO) is central to endothelial regulation. It is produced by endothelial cells and promotes smooth muscle relaxation, maintains vessel compliance, and limits vascular resistance [6,33]. Regulation of vascular tone depends on the balance between vasodilator and vasoconstrictor influences. Hypertension is characterised by impairment of endothelium dependent vasodilation together with sustained vasoconstrictor activation. Reduced NO availability limits vasodilation. Human forearm plethysmography studies have consistently demonstrated impaired acetylcholine-mediated vasodilation in essential hypertension, indicating reduced endothelial NO bioavailability [33,35]. Recent human studies consistently demonstrate that impaired nitric oxide signalling is closely linked to cardiometabolic dysfunction and increased vascular resistance [33,34].

Oxidative stress contributes through rapid scavenging of nitric oxide by superoxide, reducing NO bioavailability and increasing peroxynitrite formation [8]. Inflammatory signalling inhibits eNOS activity and promotes endothelial dysfunction [6,35,36]. The contribution of endothelial repair mechanisms remains uncertain. Reduced number and impaired function of endothelial progenitor cells (EPCs) have been reported in hypertension and are associated with vascular dysfunction and cardiovascular risk, although whether this represents a cause or consequence of endothelial dysfunction has not been established [37,38].

### 3.2. Neurohormonal Control: RAAS and Sympathetic Activation

Vasoconstrictor control is mediated primarily by the renin–angiotensin–aldosterone system (RAAS) and the sympathetic nervous system. Angiotensin II increases vascular tone and promotes sodium reabsorption in the kidney. Aldosterone enhances sodium retention and contributes to volume expansion. Sympathetic activation increases peripheral resistance and stimulates renin release, linking neural input to RAAS activation [29,30]. Neurohormonal pathways sustain this disturbance by maintaining a cycle of vasoconstriction and oxidative stress within the vascular wall. Angiotensin II is associated with increased oxidative stress, further impairing endothelial function [6]. Sex differences in RAAS activity have been reported. Experimental and clinical studies suggest that sex hormones influence components of the RAAS, although the precise mechanisms and their relative importance in humans remain incompletely defined [13,15].

Overall, current evidence supports the presence of sex-related variation in RAAS regulation, although the extent to which these differences translate into distinct clinical phenotypes is not fully resolved.

### 3.3. Renal Sodium Handling

Renal regulation maintains elevated blood pressure over time. Angiotensin II promotes sodium reabsorption in the proximal tubule, while sympathetic activation stimulates renin release and further enhances sodium retention [39,40]. These processes reduce the effectiveness of pressure natriuresis, which is considered a key mechanism in long term blood pressure regulation [39]. The interaction between renal sodium handling and vascular resistance contributes to sustained hypertension. 

Sex hormones modulate renal sodium handling and influence key transport processes within the kidney [15]. In humans, clinical observations indicate that salt sensitivity of blood pressure may increase after menopause [41], although the underlying mechanisms are not fully established.

### 3.4. Oestrogen and Vascular Tone

Sex differences in vascular tone are not simply a matter of oestrogen being present or absent but reflect the integrated balance of multiple hormonal and haemodynamic influences. Oestrogen has been shown to enhance endothelial nitric oxide signalling and reduce oxidative stress, supporting vasodilatory function [13,15]. With declining oestrogen levels, this endothelial support is reduced, and vascular regulation becomes increasingly dependent on vasoconstrictor signalling and sodium-retaining mechanisms. Vascular tone, therefore, reflects the integrated activity of endothelial, neurohormonal, and renal systems acting on vascular smooth muscle. Hypertension develops when impaired endothelial vasodilation is insufficient to counter sustained vasoconstrictor signalling and renal sodium retention, resulting in persistent elevation of peripheral resistance and arterial pressure.

In premenopausal women, oestrogen supports vasodilatory tone through at least three parallel mechanisms. First, it activates membrane oestrogen receptors (ERα) on endothelial cells, stimulating eNOS via the PI3K/Akt pathway and increasing NO bioavailability independently of transcriptional changes [42]. Second, it suppresses NADPH oxidase activity, reducing the superoxide-driven degradation of NO that characterises oxidative endothelial stress [8]. Third, it modulates sympathetic outflow centrally: oestrogen replacement in ovariectomised rats reduces oxidative stress in the rostral ventrolateral medulla, a brainstem region that sets basal sympathetic tone [30,43], and transdermal oestrogen in postmenopausal women has been shown to decrease the rate of sympathetic nerve discharge compared with untreated controls [44]. These mechanisms do not act independently; NO itself modulates sympathetic neurotransmission at the vascular wall [6], and reduced NO availability therefore amplifies adrenergic vasoconstriction. The result is that intact oestrogen signalling maintains a vascular environment in which vasodilator and vasoconstrictor influences are balanced toward lower resting resistance. This illustrates how oestrogen affects endothelial function, sympathetic activity, and RAAS signalling together, supporting the concept that hormonal state influences vascular regulation at multiple levels rather than through a single pathway [13,15].

The transition to menopause disrupts this balance across all three regulatory levels simultaneously. Reduced NO production, increased oxidative degradation of residual NO, and heightened sympathetic drive converge to elevate peripheral vascular resistance. Evidence from microneurography studies confirms that muscle sympathetic nerve activity (MSNA) is significantly higher in postmenopausal compared with premenopausal women, and that this increase occurs more rapidly with age in women than in men after the fourth decade [45]. Critically, these changes interact: sympathetic activation stimulates RAAS, RAAS-derived angiotensin II drives oxidative stress via NADPH oxidase, and oxidative stress impairs endothelial NO synthesis, contributing to reinforcing interactions that may be less effectively counterbalanced following menopause [6,46]. This is not simply the removal of a protective factor; rather, it reflects the concurrent dysregulation of three coupled regulatory mechanisms previously maintained in balance by oestrogen. Sex-specific modulation of vascular tone reflects the coordinated action of oestrogen across endothelial, neurohormonal, and renal systems. The menopausal transition removes this coordination, and the resulting dysregulation at each level mutually reinforces the others, producing a rate and magnitude of blood pressure rise that cannot be accounted for by any single mechanism in isolation.

### 3.5. Androgens and Vascular Tone

Oestrogen withdrawal is well recognised as an important contributor to postmenopausal hypertension, but the concurrent shift in androgen status also contributes to vascular dysfunction. In postmenopausal women, declining sex hormone-binding globulin is associated with increased free testosterone availability [47]. Animal studies demonstrate that testosterone upregulates the vasoconstrictive ACE/angiotensin II/AT1R axis, opposing the protective shifts promoted by oestrogen [15,40], and data from the Multi Ethnic Study of Atherosclerosis demonstrate that a higher testosterone to oestradiol ratio is independently associated with increased risk of cardiovascular events and incident hypertension in postmenopausal women [48]. The net hormonal environment of the postmenopausal vasculature therefore reflects not only oestrogen deficiency but a concurrent shift in the androgen to oestrogen balance that contributes to vascular dysfunction [13,15]. The relative contributions of oestrogen withdrawal versus androgen excess to postmenopausal hypertension remain incompletely resolved but have important therapeutic implications.

### 3.6. Genetics and Blood Pressure Regulation

Genetics plays an important role in determining individual risk of developing hypertension [49]. Large genome-wide association studies have established that blood pressure is a complex, polygenic trait influenced by hundreds of loci; a landmark analysis of over one million individuals of European ancestry identified 535 new loci linked to systolic and diastolic pressures and hypertension risk [32]. Many of these loci are located near genes involved in vascular smooth muscle function, endothelial function, renal sodium handling, and neurohormonal regulation [50]. Genetic susceptibility to hypertension does not operate independently of hormonal regulation. Many genetic variants associated with blood pressure influence pathways involved in the renin–angiotensin–aldosterone system, sympathetic nervous system activity, and renal sodium handling. This suggests that inherited blood pressure risk and hormonal status interact to shape individual susceptibility to hypertension [32,50]. The rs1799945 GG genotype in *HFE* independently increased hypertension risk in women, with sex-specific differences identified across GWAS blood pressure loci and intergenic interactions amplifying susceptibility further [51,52]. Several blood pressure-associated loci, including *FGF5*, *SH2B3, MECOM, ZNF831*, and *FTO*, have also been identified as associated with preeclampsia [53]. The overlap between genetic pathways involved in chronic hypertension and hypertensive disorders of pregnancy suggests that these conditions may share common biological mechanisms. Some women may enter pregnancy with inherited susceptibility that affects vascular adaptation and becomes clinically evident when the cardiovascular demands of pregnancy increase [50,54].

## 4. Metabolic Syndrome

The rising prevalence of metabolic syndrome is a major driver of hypertension risk. Individuals with metabolic syndrome, a cluster of interrelated conditions including central obesity, insulin resistance, dyslipidaemia, physical inactivity, and chronic low-grade inflammation, are substantially more likely to develop high blood pressure over time [1,55]. Current guidelines consistently recommend lifestyle interventions including dietary sodium restriction (<5 g/day), increased physical activity, and weight management as cornerstone strategies for the prevention and management of hypertension [56,57]. Importantly, a prospective cohort study demonstrated that metabolically unhealthy individuals, even when not obese, have a markedly higher risk of developing hypertension compared to metabolically healthy individuals of normal weight [55]. These metabolic alterations interact directly with vascular regulatory systems, particularly through effects on endothelial nitric oxide signalling and inflammation [27,58].

Insulin resistance is strongly associated with an increased risk of hypertension, and elevated fasting insulin levels and reduced insulin sensitivity play a critical role in vascular health and blood pressure regulation. Under normal conditions, insulin promotes NO release via the IRS-1/PI3K signalling pathway, facilitating vasodilation, and inhibiting abnormal vascular smooth muscle cell growth, key mechanisms that reduce thrombosis and inflammation [58]. In insulin resistant states, this NO-mediated pathway is impaired, leading to endothelial dysfunction and heightened cardiovascular risk [58]. Importantly, women may be particularly susceptible to inflammation-driven endothelial dysfunction, especially following menopause; whereas, men typically present with earlier blood pressure elevations linked to visceral adiposity and sympathetic overactivity [13,14]. Reduced nitric oxide availability further limits microvascular perfusion and contributes to impaired glucose disposal and reinforcing insulin resistance [58,59]. Endothelial dysfunction is also promoted by inflammation and oxidative stress associated with hypertension, which reduces insulin sensitivity [6,27]. Through inflammation, oxidative stress, and insulin resistance, hypertension damages the endothelium and fosters a pro-atherogenic environment [27,60]. Furthermore, atherosclerosis, particularly in the renal arteries, may contribute to secondary hypertension by reducing renal perfusion and activating the RAAS, which increases systemic blood pressure. Hypertension and atherosclerosis form a vicious cycle, with hypertension promoting endothelial injury and progression of atherosclerotic disease, and advanced atherosclerosis worsening vascular dysfunction and further elevating blood pressure [61]. Collectively, these mechanisms may contribute to impaired vascular homeostasis during states of metabolic and hormonal change.

## 5. Vascular Ageing

Vascular ageing involves a range of cellular and molecular changes rather than a single underlying mechanism. Oxidative stress increases with age and is accompanied by mitochondrial dysfunction, characterised by increased mitochondrial reactive oxygen species production, reduced ATP generation, and impaired mitophagy, alongside a persistent low-grade inflammatory state often termed ‘inflammageing’ [62]. These processes promote extracellular matrix remodelling and limit vascular repair. Over time, these cumulative alterations contribute to endothelial dysfunction and drive the progressive increase in arterial stiffness observed with ageing, ultimately reflecting the combined impact of structural remodelling and impaired vascular regulation [6,9,27,58,63]. These processes represent distinct mechanisms but also modify the behaviour of the regulatory systems described in previous sections. Increased oxidative stress within ageing vasculature promotes endothelial nitric oxide synthase uncoupling and reduces nitric oxide bioavailability, while interacting with angiotensin II-mediated pathways [6], inflammatory processes contribute to metabolic dysregulation [27,58]. In particular, impaired endothelial function and increased arterial stiffness reduce arterial compliance, leading to progressive loss of vascular buffering capacity [64]. Arterial stiffness is most commonly quantified using carotid femoral pulse wave velocity (cfPWV), the gold standard non-invasive measure of central arterial stiffness and a strong independent predictor of cardiovascular events [65,66]. Inflammatory mediators and reactive oxygen species increase with age, further exacerbating endothelial dysfunction, reducing NO bioavailability and impairing vasodilation through diminished physiological activation of eNOS. Increases in asymmetric dimethylarginine (ADMA), an endogenous NOS inhibitor generated by proteolysis of methylated proteins and cleared primarily via renal and hepatic DDAH-mediated metabolism, are associated with impaired endothelial function, with elevated levels observed in hypertensive models [67]. Vascular ageing is often accompanied by progressive medial arterial calcification. This process is driven by the osteogenic transdifferentiation of vascular smooth muscle cells and the dysregulation of endogenous calcification inhibitors, such as matrix Gla protein and the osteoprotegerin/RANKL/RANK axis. This process is distinct from intimal atherosclerotic calcification and contributes to arterial stiffening, increasing cfPWV beyond the effects of transient blood pressure changes [68,69,70].

In addition to large artery stiffening, vascular ageing is associated with significant alterations in the microcirculation. Microvascular dysfunction, a recognised feature of ageing, is driven in part by structural changes in the endothelial glycocalyx. Older adults exhibit reduced glycocalyx thickness in sublingual microvessels compared to younger individuals, a finding supported by both human and experimental studies, which is associated with impaired barrier function and reduced microvascular perfusion [71]. Beyond glycocalyx degradation, microvascular dysfunction also reflects capillary rarefaction, impaired angiogenic signalling, increased oxidative stress, altered vascular smooth muscle responsiveness, and reduced endothelial progenitor cell mobilisation. Collectively, these changes elevate peripheral resistance and impair tissue perfusion, contributing to blood pressure dysregulation with ageing [9,72].

Despite the age-related decline in vascular function in both sexes, it is widely recognised that there are significant differences in hypertension risks between men and women, with men having a higher prevalence of hypertension earlier in life while women experience a sharper rise in blood pressure around and after menopause [13,31]. Moreover, patterns of vascular ageing, including changes in arterial stiffness and endothelial function, differ between the sexes, with postmenopausal women showing a more pronounced increase in arterial stiffness compared with men of similar age, contributing to sex-specific trajectories of hypertension and cardiovascular risk [13,31,63]. This is reflected in endothelial function, which demonstrates sex-specific differences across an individual’s lifespan [13,63]. Structural changes with age, including intima-media thickening, arterial diameter widening, and progressive stiffening due to slow-onset atherosclerosis, further increase arterial rigidity. Combined with greater carotid intima-media thickness, men tend to show poorer vascular function earlier in adulthood; whereas, women maintain better endothelial function into midlife. This advantage declines around menopause, with a faster deterioration in vascular function that leads to convergence and eventual reversal of cardiovascular risk later in life [73,74].

Sex differences in vascular function likely reflect a combination of ageing, angiogenesis and impaired endothelial regeneration in both men and women, largely due to diminished repair capacity and compromised microvascular maintenance [11,26]. This decline is associated with reduced secretion and responsiveness to key growth factors such as VEGF and FGF [9]. In addition, ageing promotes accumulation of senescent vascular and endothelial cells, characterised by stable cell cycle arrest (e.g., p16INK4a/p21 upregulation) and a senescence-associated secretory phenotype (SASP) that releases pro-inflammatory cytokines and matrix metalloproteinases into the vascular wall, alongside apoptotic signalling that together reduce regenerative potential and accelerate vascular dysfunction in both sexes [28]. Together, these structural and functional changes suggest that women may experience a more rapid transition in vascular ageing trajectories after midlife which may underlie the rapid increase in hypertension prevalence observed during and following the menopausal transition [73,74].

Ageing and hypertension significantly affect cerebral circulation. Hypertension is strongly associated with cerebral small vessel disease (CSVD), white matter injury, microvascular rarefaction, and disruption of blood–brain barrier integrity. These factors all contribute to impaired cerebral perfusion and cognitive decline [11,12,75]. Sex differences in cerebrovascular regulation have been increasingly recognised, with important implications for vascular cognitive impairment. During women’s midlife, declining oestrogen levels are associated with reduced nitric oxide bioavailability, increased oxidative stress, and impaired microvascular function. All of these changes contribute to compromising cerebrovascular integrity and vascular cognitive ageing. Differences in endothelial signalling, neurovascular coupling, and autoregulatory capacity may contribute to altered cerebral blood flow regulation in women, particularly during and after the menopausal transition [76].

## 6. Role of Oestrogen

Although premenopausal women generally exhibit healthier vasculature and a lower rate of hypertension than men, this advantage diminishes following menopause in association with declining oestrogen levels [9,66]. However, sex differences in hypertension cannot be attributed to oestrogen alone. Testosterone also influences endothelial function, vascular tone, and RAAS activity, and changes in the androgen to oestrogen balance across the female lifespan may further modulate blood pressure regulation. Accordingly, sex differences in hypertension emerge from the interaction between multiple hormonal systems, including sex steroids, adrenal hormones, and neuroendocrine regulation, rather than from oestrogen deficiency alone [13,73].

Of course, these hormonal effects are not isolated but interact with metabolic, autonomic, and vascular regulatory systems, such that changes in oestrogen levels influence multiple physiological pathways simultaneously rather than a single mechanism.

One of the major protective factors before menopause is oestrogen, which supports vascular health by reducing oxidative stress and inflammation, preserving endothelial function, and maintaining overall vascular integrity [6,8]. As outlined before, these protective effects operate through coordinated modulation of endothelial nitric oxide signalling, sympathetic outflow, and RAAS activity [15,42]. With declining oestrogen at menopause, these regulatory mechanisms are simultaneously disrupted, shifting vascular homeostasis toward increased peripheral resistance [39,77].

These mechanisms are summarised schematically in Figure 1, which illustrates the integrated interactions among endothelial, neurohormonal, metabolic, and ageing related pathways that contribute to hypertension across the menopausal transition.

In vitro and animal studies have demonstrated that 17β-oestradiol enhances angiogenic capacity, cell proliferation, and mobilisation of endothelial progenitor cells (EPCs), while also promoting EPC-mediated repair of blood vessels [78]. These vascular effects also extend to cerebral circulation. Oestrogen contributes to neurovascular coupling and cerebrovascular regulation, and its decline may impair microvascular integrity and increase susceptibility to vascular cognitive impairment, although causal mechanisms remain incompletely defined. These changes may reflect alterations in cerebrovascular autoregulation and microvascular function, which are critical for maintaining stable cerebral perfusion and are increasingly recognised as contributors to vascular cognitive ageing [11,63].

Whilst the decline in oestrogen influences vascular resistance through the removal of key protective mechanisms, menopause is also associated with secondary factors such as increased body mass index (BMI), anxiety, and sleep disturbances that further compromise vascular health and amplify cardiovascular risk [79,80]. 

Together, these findings suggest that hormonal status modifies vascular control at multiple levels, rather than through a single regulatory mechanism [13,29].

In ovariectomised rats, oestrogen replacement has been shown to reduce oxidative stress markers in central autonomic regions [43], highlighting the role of oestrogen loss in promoting oxidative stress and sympathetic activation that contribute to hypertension. Women undergoing surgical menopause have a prevalence of salt sensitivity of blood pressure that increases following menopause. This is mainly a result of oestrogen deficiency altering the balance between RAAS activity and nitric oxide signalling and disrupting renal sodium handling [41]. Therefore, alongside vascular ageing, the risk of hypertension is accelerated by the loss of oestrogen’s protective effects following menopause, including heightened RAAS sensitivity, increased sympathetic activity, reduced endothelial function, and greater oxidative stress, consistent with the integrated vascular framework illustrated in Figure 1.

In addition to the natural ageing process and the decline in oestrogen, additional factors influence vascular function and the onset of hypertension in women. Sleep disruption is common during the menopausal transition and has been associated with adverse cardiovascular health [81]. In a cross-sectional pilot study of postmenopausal women aged 50–74 years, poor sleep quality was independently associated with higher central arterial stiffness, as measured by cfPWV and baPWV, even after adjusting for age, blood pressure, and physical activity [79].

Sleep disturbances are associated with alterations in HPA axis activity and cortisol patterns, which may contribute to elevated cardiovascular risk [81]. These changing levels of cortisol are associated with heightened stress and anxiety [82]. Longitudinal analyses in the Seattle Midlife Women’s Health Study showed that differences in reported sleep difficulties across the menopausal transition were linked to first morning urine cortisol levels [81]. Changes in cortisol levels are associated with increased cardiometabolic risk and aggravate metabolic symptoms, such as reduced insulin sensitivity, which, combined with heightened sympathetic stimulation and impaired glucose metabolism, promote metabolic syndrome [1,30].

The accumulation of visceral fat, which rises significantly after menopause, is also strongly linked to hypertension and cardiometabolic disease [80]. Increased adiposity, compounded by hormonal dysregulation, will accelerate vascular ageing and promote remodelling of the vascular extracellular matrix, leading to reduced arterial compliance and increased systolic blood pressure [72,81]. Therefore, a combination of sleep deprivation, sustained cortisol elevation, and weight gain is associated with a cascade of metabolic and vascular changes that markedly increase hypertension risk in postmenopausal populations [1,30,80].

### Hormone Therapy: Clinical Evidence and the Timing 

The cardiovascular effects of exogenous oestrogen in postmenopausal women cannot be interpreted independently of large scale randomised clinical trial evidence. The Women’s Health Initiative (WHI) hormone therapy trials initially reported increased risks of coronary heart disease, stroke, and venous thromboembolism in women receiving conjugated equine oestrogen combined with medroxyprogesterone acetate, particularly in older postmenopausal women with a mean age of approximately 63 years [83]. These findings led to early concerns that hormone therapy may confer net cardiovascular harm. However, interpretation of these findings requires distinguishing between effects on blood pressure and broader cardiovascular outcomes, as large clinical trials have primarily evaluated clinical events rather than isolated changes in vascular function.

Subsequent re-analyses and extended follow up data have substantially refined this interpretation through the development of the “timing hypothesis.” Evidence indicates that cardiovascular outcomes are strongly dependent on the interval between menopause onset and initiation of hormone therapy. Women who initiate hormone therapy within approximately ten years of menopause, or before the age of 60, do not exhibit an increased risk of coronary heart disease and, in some analyses, demonstrate a risk reduction [22,23,83]. This pattern is physiologically coherent with endothelial biology: oestrogen mediated effects on nitric oxide bioavailability, oxidative stress suppression, and modulation of renin–angiotensin system activity is most effective in vessels with preserved endothelial structure and function [6,8,42]. In contrast, when advanced atherosclerotic remodelling is already established, the same hormonal environment may no longer restore vascular homeostasis and may interact differently with plaque stability and vascular inflammation [27].

The cardiovascular effects of hormone therapy are further modified by formulation and route of administration. Transdermal oestradiol avoids first-pass hepatic metabolism and is associated with a lower risk of venous thromboembolism and a more favourable haemostatic profile compared with oral preparations [24,83]. In addition, micronised progesterone appears to exert a more neutral vascular and thrombotic profile than synthetic progestogens such as medroxyprogesterone acetate [83].

Glucagon-like peptide-1 (GLP-1) receptor agonists and sodium glucose cotransporter 2 (SGLT2) inhibitors have demonstrated significant cardiovascular benefit in large clinical trials, including reductions in major cardiovascular events and mortality [84]. These therapies are also associated with improvements in endothelial function and enhanced nitric oxide bioavailability, as well as favourable effects on body weight, insulin sensitivity, and blood pressure [84]. These therapies may influence multiple components of the integrated vascular metabolic framework described in this review. However, further research is required to determine how they can be incorporated into sex-specific approaches to hypertension management.

Other targeted approaches, including selective oestrogen receptor modulators and endothelin receptor antagonists, may also influence blood pressure through their effects on vascular tone and endothelial function, although their role in female-specific hypertension remains incompletely defined [85,86].

## 7. Hypertensive Disorders of Pregnancy

Hypertensive disorders of pregnancy (HDP), including gestational hypertension and preeclampsia, are not simply obstetric complications that resolve after delivery. Different forms of HDP are associated with increased cardiovascular disease risk, although the magnitude and pattern of outcomes vary between subtypes [87,88]. These associations may reflect both pregnancy-related vascular changes and pre-existing cardiovascular susceptibility. HDP may therefore act as female specific markers of future cardiovascular risk by identifying women with increased susceptibility to hypertension and cardiovascular disease later in life [87]. Preeclampsia is the most extensively studied HDP subtype in relation to long-term cardiovascular outcomes [16]. Women with a history of preeclampsia are four times more likely to develop heart failure and twice as likely to experience coronary heart disease, stroke, and cardiovascular death [16]. They also demonstrate insulin resistance and increased circulating endothelial cells, supporting the presence of metabolic disturbance and endothelial injury in the condition [25]. Structural cardiac remodelling has been documented in women with preeclampsia at term, suggesting early cardiovascular alterations that may contribute to later cardiovascular vulnerability [26]. Preeclampsia may reflect underlying cardiovascular susceptibility, as it is associated with an increased risk of future cardiovascular disease [89]. Figure 1 illustrates how several of the vascular and metabolic pathways discussed earlier in this review are also implicated in the pathophysiology of preeclampsia.

### Genetic Susceptibility

Genome wide association studies (GWAS) have identified shared genetic loci between blood pressure traits and preeclampsia susceptibility in European and Central Asian populations [53]. Several genes involved in blood pressure regulation, including *FGF5*, *SH2B3*, *MECOM*, *ZNF831* and *FTO*, show associations with preeclampsia [53]. *PLEKHG1* encodes a Rho GTPase exchange factor involved in regulation of the endothelial cytoskeleton. It has also been linked to an increased susceptibility to preeclampsia [90]. Also, essential hypertension and preeclampsia share considerable genetic architecture, and studying their overlap may help clarify common biological mechanisms [91].

A genetic predisposition to higher diastolic blood pressure has been associated with an increased likelihood of preeclampsia, independent of conventional cardiovascular risk factors [92]. However, genetic risk scores based on established blood pressure and hypertension loci were not associated with preeclampsia in two independent cohorts [93]. The rs1799945 GG genotype in *HFE* independently increased preeclampsia risk and nine out of ten hypertension loci showed epistatic interactions. This clearly highlights the role of gene–gene interactions in preeclampsia susceptibility [94]. Therefore, genetic overlap between hypertension and preeclampsia is specific to certain blood pressure traits rather than reflecting a broad shared genetic basis. Pre-pregnancy body mass index (BMI) may further influence this genetic susceptibility. In overweight and obese women, rs167479 (*RGL3*) was associated with an increased likelihood of preeclampsia, whereas rs805303 (*BAG6*) was associated with a reduced risk [95].

Genetic variants involved in blood pressure regulation have also been examined in relation to changes in maternal blood pressure during pregnancy [96,97]. The 4a4a genotype of the endothelial nitric oxide synthase (eNOS) VNTR polymorphism has been associated with higher diastolic blood pressure towards the end of pregnancy. Among women carrying this genotype, women with higher pre-pregnancy BMI show greater increases in systolic, diastolic, and mean arterial pressure during late gestation [97]. The angiotensin-converting enzyme (ACE) insertion/deletion polymorphism has also been associated with elevated blood pressure in late pregnancy [96]. The findings suggest that the vascular dysfunction associated with preeclampsia is influenced by genetic susceptibility that exists prior to conception. Table 1 summarises the key genetic studies.

## 8. Conclusions

Hypertension in women does not result from a single mechanism. Rather, it develops over time through changes in neurohormonal regulation and vascular function that are influenced by hormonal and metabolic factors. These changes contribute to endothelial dysfunction, increased sympathetic activity, and progressive increases in blood pressure. The menopausal transition is accompanied by reduced nitric oxide bioavailability, impaired endothelial function, altered RAAS responsiveness, and a shift towards vasoconstrictor influences, all of which contribute to vascular dysfunction [13].

Hypertension is also known to disrupt the blood–brain barrier, causing cerebrovascular dysfunction and cognitive decline [11,12].

Women with a history of hypertensive disorders of pregnancy may be particularly vulnerable to these changes. Persistent vascular dysfunction and reduced endothelial reserve can continue long after pregnancy and are associated with increased long-term cardiovascular risk [25]. As additional physiological stressors accumulate with age, the capacity to maintain vascular homeostasis may be reduced, lowering the threshold for the development of sustained hypertension.

Although many mechanisms contributing to hypertension in women have been extensively investigated, how they interact remains unclear. Future research should focus on longitudinal studies across the menopausal transition to improve understanding of these interactions across the female life course. Improved integration of reproductive history, menopausal status, and vascular risk assessment may facilitate earlier identification of women at increased cardiovascular risk and support more personalised prevention strategies.

Hypertension in women has a heritable component. Some blood pressure loci overlap with preeclampsia susceptibility, pointing to shared inherited vascular mechanisms that may operate across the female life course [32,53]. The strength and direction of these associations vary across loci, and gene–gene interactions further complicate how genetic risk translates into clinical disease [51,52]. Improved integration of reproductive and sex-specific risk factors into cardiovascular risk assessment may facilitate earlier identification of women at increased cardiovascular risk and support more personalised prevention strategies. This approach is consistent with recent ESC and AHA/ACC guidance recognising the importance of these factors in cardiovascular prevention and risk assessment [98,99].

## Figures and Tables

**Table 1:** Genetic factors associated with blood pressure regulation and preeclampsia susceptibility during pregnancy.

Study	Genetic Factor/Locus	Key Finding
Steinthorsdottir et al. (2020) [53]	*FGF5*, *SH2B3*, *MECOM*, *ZNF831*, *FTO*	GWAS in European and Central Asian populations identified genome-wide significant maternal associations with preeclampsia at *ZNF831*/20q13 and *FTO*/16q12; further analysis identified sub-threshold associations at *MECOM*/3q26, *FGF5*/4q21, and *SH2B3*/12q24; a polygenic risk score for hypertension was also independently associated with preeclampsia, demonstrating that genetic predisposition to hypertension is a risk factor for the condition
Gray et al. (2018) [90]	*PLEKHG1*	Identified *PLEKHG1* as a novel maternal susceptibility locus for preeclampsia, encoding a Rho GTPase exchange factor involved in endothelial cytoskeletal organisation
Gray et al. (2021) [92]	Genetic instruments for diastolic blood pressure and BMI	Genetic predisposition to higher diastolic blood pressure was associated with increased preeclampsia risk, with the effect stronger for early-onset preeclampsia below 34 weeks (OR 1.30) than overall (OR 1.11); genetic predisposition to higher BMI was also independently associated with preeclampsia risk (OR 1.10)
Smith et al. (2016) [93]	Genetic risk scores for hypertension, SBP, DBP, and MAP	Genetic risk scores across all four blood pressure traits showed no significant association with preeclampsia risk in the discovery cohort, with null findings replicated in an independent cohort
Churnosov et al. (2022) [94]	*AC026703.1* rs1173771, *HFE* rs1799945, *BAG6* rs805303, *PLCE1* rs932764, *OBFC1* rs4387287, *ARHGAP42* rs633185, *CERS5* rs7302981, *ATP2B1* rs2681472, *TBX2* rs8068318, *RGL3* rs167479	The *HFE* rs1799945 GG genotype independently increased preeclampsia risk (OR 2.24); *BAG6* rs805303 was associated with reduced preeclampsia risk (OR 0.55–0.78); nine of ten SNPs appeared in the ten most significant epistatic interaction models, indicating that gene–gene interactions contribute substantially to preeclampsia susceptibility
Abramova et al. (2022) [95]	*BAG6* rs805303 and *RGL3* rs167479	In overweight and obese women, *BAG6* rs805303 was associated with reduced preeclampsia risk (OR 0.36–0.66) and *RGL3* rs167479 was associated with increased preeclampsia risk (OR 1.86); neither variant was associated with preeclampsia in normal-weight women, demonstrating that pre-pregnancy BMI modifies the expression of blood pressure genetic risk
Reshetnikov et al. (2019) [97]	eNOS VNTR polymorphism (4a/4b)	The eNOS 4a4a genotype was associated with higher diastolic blood pressure at the end of pregnancy; among 4a4a carriers with elevated pre-pregnancy BMI, systolic, diastolic, and mean arterial pressures were higher at that stage of gestation
Reshetnikov et al. (2015) [96]	ACE insertion/deletion polymorphism	The ACE insertion/deletion polymorphism was associated with elevated blood pressure at the end of pregnancy

Abbreviations: GWAS, genome-wide association study; SNP, single nucleotide polymorphism; SBP, systolic blood pressure; DBP, diastolic blood pressure; MAP, mean arterial pressure; BMI, body mass index.

## Data Availability

No new data was created or analysed in this study. Data sharing is not applicable to this article.

## Abbreviations

The following abbreviations are used in this manuscript:

ACE	Angiotensin-converting enzyme
ADMA	Asymmetric dimethylarginine
AT1R	Angiotensin II type 1 receptor
baPWV	Brachial-ankle pulse wave velocity
cfPWV	Carotid-femoral pulse wave velocity
eNOS	Endothelial nitric oxide synthase
EPC	Endothelial progenitor cell
ERa	Oestrogen receptor alpha
FGF	Fibroblast growth factor
HDP	Hypertensive disorders of pregnancy
HPA	Hypothalamic–pituitary–adrenal
HRT	Hormone replacement therapy
IRS-1	Insulin receptor substrate-1
MESA	Multi-Ethnic Study of Atherosclerosis
MSNA	Muscle sympathetic nerve activity
NADPH	Nicotinamide adenine dinucleotide phosphate
NO	Nitric oxide
NOS	Nitric oxide synthase
PI3K	Phosphoinositide 3-kinase
PSQI	Pittsburgh Sleep Quality Index
RAAS	Renin–angiotensin–aldosterone system
ROS	Reactive oxygen species
SHBG	Sex hormone-binding globulin
SWAN	Study of Women’s Health Across the Nation
VEGF	Vascular endothelial growth factor
WHI	Women’s Health Initiative
